# Central American Immigrant Parents’ Awareness, Acceptability, and Willingness to Vaccinate Their Adolescent Children Against Human Papillomavirus: A Pilot Cross-Sectional Study

**DOI:** 10.3390/ijerph17082869

**Published:** 2020-04-21

**Authors:** Ana Cristina Lindsay, Joanna A. Pineda, Madelyne J. Valdez, Maria Idalí Torres, Phillip J. Granberry

**Affiliations:** 1 Department of Exercise and Health Sciences, College of Nursing and Health Sciences, University of Massachusetts Boston, Boston, MA 02125, USA; Joanna.Pineda001@umb.edu (J.A.P.); Madelyne.Valdez001@umb.edu (M.J.V.); 2 Department of Anthropology, College of Liberal Arts, University of Massachusetts Boston, Boston, MA 02125, USA; idali.torres@umb.edu; 3 The Mauricio Gastón Institute for Latino Community Development and Public Policy, University of Massachusetts Boston, Boston, MA 02125, USA; phillip.granberry@umb.edu; 4 Economics Department, College of Liberal Arts, University of Massachusetts Boston, Boston, MA 02125, USA

**Keywords:** human papillomavirus, HPV, HPV vaccine, Central American, Latino, immigrant, parents, exploratory, community-based

## Abstract

Despite increasing interest in understanding the factors influencing awareness and acceptability of the human papillomavirus (HPV) vaccine among Latino parents, to date limited information is available specific to Central American parents living in the United States (US). Therefore, this pilot cross-sectional study was designed to explore and assess Central American immigrant parents’ awareness, acceptability, and willingness to vaccinate their children against HPV, and interest in participating in future HPV-associated cancer prevention study. Fifty-six Central American parents, majority immigrant (96.4%; *n* = 54) from four countries, El Salvador—50% (*n* = 27); Guatemala—25.9% (*n* = 14); Honduras—22.2% (*n* = 12); and Panama—1.9% (*n* = 1) participated in this study. Participants completed an interviewer-administered questionnaire survey in their preferred language (i.e., Spanish or English). A little over half of the participants were mothers (57.1%; *n* = 32) and parents’ mean age was 43.2 years (SD = 6.4). The majority was married or cohabitating (76.8%, *n* = 43), and 39.3% (*n* = 22) reported having two children. Seventy-five percent (*n* = 42) of parents reported they had heard of the HPV vaccine. Fewer fathers were aware of the HPV vaccine (58.3%; *n* = 14 vs.87.5%, *n* = 28; *p* = 0.01) than mothers. Among parents who had heard of the HPV vaccine (*n* = 42), 85.7% (*n* = 36) reported their children had received at least one dose of the HPV vaccine. Fewer fathers reported their child had been vaccinated against HPV (64.3%, *n* = 9 vs. 96.4%, *n* = 27; *p* = 0.06) than mothers. Moreover, 90% of parents (*n* = 18) whose children were unvaccinated reported willingness to vaccinate their adolescent children against HPV if recommended by their child’s physician. Findings indicate parents’ low to moderate awareness of the HPV vaccine, and high willingness to vaccinate their adolescent children if recommended by their child’s physician. Findings also demonstrate fathers’ lower awareness and acceptability of the HPV vaccine than mothers. Despite limitations and the need for more research, findings of this pilot study serve as a valuable first step toward building a knowledge foundation that is needed for developing future studies and interventions targeting Central American immigrant parents living in the US. Future studies can build on the findings of this exploratory study with other research designs and address its limitations by having a larger sample size and accounting for additional factors associated with Central American immigrant parents’ HPV awareness, knowledge, beliefs, attitudes, and vaccine acceptability from other communities across the US.

## 1. Introduction

Latinos or Hispanics (hereafter referred to as Latinos) are the largest and most rapidly growing minority group in the United States (US) [1]. The current US Latino population is drawn from an increasingly diverse mix of countries. Evidence suggests that Central American countries (e.g., El Salvador, Guatemala, Honduras, etc.) account for a rapidly increasing proportion of the immigrant Latino population in the US [2,3]. According to the American Community Survey from 2008 to 2018, the Central American child population increased by 55%, while the total Latino child population increased by 10% [4].

Cancer is the leading cause of death among Latinos in the US, surpassing cardiovascular disease [5,6], thus making cancer prevention and control, a pressing public health priority. Although data indicate that Latinos have lower incidence rates than non-Hispanic whites for the most common type of cancers, it is estimated that 30% of Latino men and Latina women will be diagnosed with cancer during their lifetime [6,7].

Genital HPV is the most common sexually transmitted infection in the US, and an estimated 14 million persons are newly infected with HPV every year [5]. HPV is etiologically linked to several types of cancers including the cervix, vulva, vagina, penis, anus, and oropharynges [7,8]. Evidence indicates that Latinos have higher HPV-associated cancer incidence and mortality than non-Hispanic whites [7,8]. For example, Latinas disproportionally experience high cervical cancer incidence and mortality rates compared to non-Hispanic whites [5,6,7]. Latino men have a penis cancer rate of 1.3 for 100,000 men, compared to 0.7 per 100,000 men of all races [5,6,7].

Approximately 150 HPV types have been identified, and over 40 of which infect the genital area [8,9]. Genital HPV types are categorized according to their epidemiologic association with cancer risk—“high-risk” (cancer-causing) or “low-risk” (wart-causing) [8,9]. High-risk types (e.g., types 16 and 18) have the potential to act as carcinogens, while low-risk types (e.g., types 6 and 11) can cause benign or low-grade cervical cell changes, genital warts, and recurrent respiratory papillomatosis [8,9].

HPV-associated cancers can be prevented, and research shows that the most efficient and cost-effective mechanism for combating HPV is to prevent infection through vaccination [8,9,10]. Currently, three vaccines, a bivalent HPV vaccine (HPV2) called Cervarix^®^ (GlaxoSmithKline, London, United Kingdom) and two quadrivalent HPV vaccines (HPV4), called Gardasil^®^ and Gardasil^®^ 9 (Merck & Co., Kenilworth, NJ, USA) are licensed and approved for use by the US Food and Drug Administration (FDA) for prevention of several HPV-associated cancers [9,10,11]. Both the HPV2 and the HPV4 vaccines protect against HPV types 16 and 18, which cause 70% of cervical cancers [9,10,11]. HPV type 16 also causes the majority of other cancers attributable to HPV, and the HPV4 vaccines also protects against HPV types 6 and 11, which cause >90% of genital warts and recurrent respiratory papillomatosis [11].

The Advisory Committee on Immunization Practices (ACIP) and the American Academy of Pediatrics (APA) recommend routine vaccination with quadrivalent (HPV4) or bivalent (HPV2) HPV vaccine for females aged 11 or 12 years and with HPV4 for males aged 11 or 12 years [11]. Vaccination also is recommended for females aged 13 through 26 years and for males aged 13 through 21 years who were not vaccinated previously [11]. Although the ACIP began recommending routine HPV vaccination for females in the US in 2006 [11], routine vaccination was not recommended for males until 2011 [10,11]. Furthermore, although HPV vaccination is recommended, it is not mandatory, and only a few states have enacted policies for HPV vaccine school-entry requirements [10,11].

The Healthy People 2020 objectives call for an increase to 80% in the percentage of female and male adolescents aged 13 through 15 years who receive 2 or 3 doses of HPV vaccine as recommended [12]. Nonetheless, uptake of the HPV vaccines is significantly lower than other recommended adolescent vaccines [13]. According to National Immunization Survey–Teen (NIS-Teen), an annual survey conducted among parents and guardians that estimates vaccination coverage among adolescents aged 13–17 years in the US, in 2018, only 51.1% of these adolescents were up to date with the HPV vaccine series, and only 68.1% had received ≥1 dose of HPV vaccine [13]. A number of factors may account for the low HPV vaccination rates including a continued stigma related to the HPV being sexually transmitted, parents not being appropriately educated about the vaccine's safety, efficacy or necessity, barriers due to lack of or inadequate access to healthcare, vaccine cost (e.g., not free, high cost of deductible), social norms, etc. [14,15,16,17]. Furthermore, evidence suggests that physicians may not be adequately recommending vaccination at appropriate ages [18].

Despite an increasing number of studies conducted to identify knowledge and awareness of HPV and the HPV vaccine among various Latino groups in the US [19,20,21,22,23,24,25,26,27], to date, limited information specific to Central Americans exists. In fact, most of the available literature has considered Latinos as a homogeneous group [21,22,23,24], with limited published research and data specific to Central American immigrant parents living in the US. Moreover, there is a dearth of research on Latino fathers [28]. In general, the extant literature has focused on mothers, with a few studies including both mothers and fathers [14,15,16,17,21,22,23,24,25,26], and almost none focused exclusively on Latino fathers [25]. 

Findings from a recent systematic review conducted to identify studies exploring HPV knowledge, awareness, beliefs, attitudes, and acceptability of the HPV vaccine among Latino fathers living in the US identified only 11 eligible studies [27]. Furthermore, fathers’ representation in these studies was considerably low (from 8.3% to 31.9% of the sample) compared to mothers’ representation (from 68.1% to 91.7%) [28]. Of the 11 studies identified, only one was conducted exclusively with Latino fathers [24], whereas the other 10 studies that included both parents, and only two explicitly compared outcomes for fathers and mothers [14,15,16,17,20,21,22,23,24,26,27].

Findings of the only study with a national sample of Latino fathers, most of whom were foreign-born (the majority were from Mexico), showed that although almost half were aware of HPV, knowledge of the HPV vaccine was low, and knowledge gaps related to transmission and risky behaviors existed [25]. Likewise, findings of the two studies [21,26] that compared fathers’ and mothers’ HPV and HPV vaccine awareness, knowledge and uptake of the HPV vaccine determined that fathers had lower awareness of HPV and lower HPV vaccine-related knowledge than mothers [21,26]. For example, one of the two studies, a cross-sectional study conducted in Utah, found that fathers’ awareness of the HPV and the HPV vaccination were significantly lower compared to mothers [21]. In addition, the second study, a cross-sectional survey conducted with Puerto Rican parents to identify correlates of HPV vaccine initiation found that fathers were 88% less likely than mothers to report that their sons had initiated the HPV vaccine regimen [26]. Taken together results of these three studies [21,25,26] indicate the need for culturally and linguistically appropriate educational efforts to increase awareness and knowledge of HPV and the HPV vaccine among Latino fathers. In addition, the dearth of studies on Latino fathers found by this review suggests that additional research focusing on Latino fathers is needed [27].

Given the lack of studies among Central American immigrant parents living in the US, the importance of the family in the Latino culture, and the joint role parents have in health care decision-making for their children, understanding awareness and acceptability of the HPV vaccine of Central American immigrant fathers and mothers will likely make it possible to design culturally tailored interventions to meet the specific needs of this ethnic group, and to increase vaccine uptake among their children [19,23]. Therefore, the purpose of this pilot study was to explore and assess Central American immigrant: parents’: (1) awareness of HPV and the HPV vaccine; (2) acceptability of the HPV vaccine; (3) sources of information about HPV vaccination; (4) willingness to vaccinate their age-eligible adolescent children against HPV; and (5) interest in participating in a future HPV-related cancer prevention study.

## 2. Materials and Methods 

### 2.1. Study Design, Setting and Sample

The present study was conducted as part of a large ongoing, mixed-methods HPV research with multi-ethnic Latino parents [29]. The current study was an exploratory, pilot study with an understudied population conducted as a formative first step to more clearly define research questions, possible analyses, and future and larger research designs [30]. A brief cross-sectional survey was conducted between June and October 2019 in selected communities in two states: (1) Massachusetts (MA; Chelsea, East Boston, Revere), and (2) Rhode Island (RI; Central Falls and Providence) with large Central American populations.

### 2.2. Ethics, Consent, and Permissions

Our study protocol was reviewed and approved by the University of Massachusetts Boston Institutional Review Board (IRB # IRB#2017221). Written informed consent was obtained from all parents participating in this study. Parents were eligible to participate if they: (a) self-identified as Central American; (b) had at least one child aged 11–19 years; (c) lived in MA or RI; and (d) had resided in the US for at least 12 months. We recruited participants using strategies successfully employed in our previous studies with similar Latino populations, and these included: (1) posting flyers inside local Latino businesses, and community-based social and health services agencies, and (2) making announcement and posting flyers at predominantly Spanish-speaking churches [30,31,32,33,34]. Interested individuals spoke to study staff at church events or called the telephone number listed on the flyers disseminated at local Latino businesses, community-based social and health services agencies, and churches. Participants also were recruited through social networking, a “word of mouth” or snowball sampling approach. This method involved two strategies: (1) leveraging the personal and community networks of the research staff to identify potential eligible participants and then recruit them for the study [33], and (2) asking early enrollees to recommend their Latino friends who met the study eligibility criteria [35]. All interested individuals were screened in-person or via telephone by study staff. Those who met the eligibility criteria were invited to participate in the study. 

### 2.3. Data Collection and Survey Measures

Data collection followed the same procedures used in our previous HPV research with Brazilian immigrant parents and included two-sequential steps [29]. First, a trained bilingual, bicultural interviewer read and obtained written informed consent from eligible participants. Following, parents completed a brief interviewer-administered questionnaire survey in their preferred language (Spanish or English). 

The brief questionnaire survey instrument included questions adapted from previous research with Latino groups in the US that assessed: (1) participants’ awareness of HPV and the HPV vaccine, (2) sources of information about HPV vaccination, (3) acceptability of the vaccine and willingness to vaccinate their adolescent children against HPV, and (4) participants’ interest in participating in a future HPV-related cancer prevention [21,22,25,26]. In addition, the questionnaire survey assessed participants’ demographic characteristics and acculturation levels.

#### 2.3.1. Awareness of HPV and the HPV Vaccine

Participants responded to queries concerning their awareness of HPV (“Have you heard of the human papillomavirus or HPV or genital warts?”) and the HPV vaccine (“Have you heard of the HPV vaccine, also known as Gardasil or Cervarix?”). Only those who had heard of HPV were asked if they were aware of the HPV vaccine.

#### 2.3.2. Sources of Information About the HPV Vaccine

Participants who reported being aware of the HPV vaccine were asked to report where they heard about the vaccine and had multiple choices for responses including: (1) healthcare sources—physician, nurses, other healthcare professional, public health campaigns; (2) personal sources (i.e., friends and family); and (3) media sources (i.e., television, radio, billboards, the internet, social media (Facebook, Instagram, etc.); and (4) other (specify). 

#### 2.3.3. Acceptability of the HPV Vaccine and Willingness to Vaccinate

Participants who reported hearing about the HPV vaccine were also asked whether their children aged 11-19 years had received the HPV vaccination (yes, no, unsure/don’t know). In addition, parents who reported their children had not received the HPV vaccine or were unsure, were asked whether they would be willing to vaccinate against HPV if recommended by their child’s physician (yes, no, unsure/don’t know).

#### 2.3.4. Participation in Future HPV-Related Cancer Prevention Study

Finally, participants were also asked whether they would be interested in participating in future HPV-related cancer prevention study (yes, no, unsure/don’t know, refuse to respond).

#### 2.3.5. Demographics, Access to Healthcare and Acculturation Level

Participants reported their age, marital status, number of children, country of origin, years of residency in the US, primary language, educational attainment (less than high school (HS) diploma/general education degree (GED)/high school graduation/GED or higher), and annual household income (<US$40,000/≥US$40,000). In addition, participants reported if they had regular access to a healthcare provider (yes, no) and if they had health insurance (no, yes, private or government-sponsored).

Acculturation was measured using the Short Acculturation Scale for Hispanics (SASH), a 12-item scale validated for use in Latino populations [36]. The SASH assesses language use, media use, and ethnic social relations, and has good reliability (Cronbach’s alpha reliabilities 0.92–0.89 for the overall SASH scale, 0.89 for language use, 0.88 for media preference, and 0.72 for ethnic and social relations) [36,37]. An acculturation score was computed for each participant by averaging across 12 items, measured on a scale of 1 to 5. The scale developers recommend an average of ≥2.99 as the cut-point scores equal to or above this point represent higher levels of acculturation and scores below this point represent lower levels of acculturation [36]. After completion of informed consent, the brief interviewer-based questionnaire survey lasted approximately10 minutes.

### 2.4. Data Analysis

Our core data analysis approach was similar to that utilized in our prior research with Brazilian immigrant parents [29]. Data were entered in Excel and analyzed using IBM SPSS 19.0 software (Chicago, IL, USA). Frequencies were calculated for socio-demographic variables, awareness of HPV and the HPV vaccine, sources of information about the HPV vaccine for those who had heard of the vaccine, acceptability of the HPV vaccine, and willingness to vaccinate their adolescent children against HPV for parents that reported their children had not been vaccinated. Parents’ responses to the questions were compared using Chi-square test to assess significant differences by parents’ gender, acculturation score, educational attainment and household income. In addition, Fisher’s exact test was used for cells with sample size less than 5. Statistical significance for these comparisons was based on a level of 0.05.

## 3. Results

### 3.1. Participants’ Characteristics

The sample included 56 Central American parents, representing 56 unique families. All participants chose to complete the questionnaire survey in Spanish. As shown in Table 1, a little over half of the participants were mothers (57.1%; *n* = 32); and parents’ mean age was 43.2 years (SD = 6.4).

The majority was married or cohabitating (76.8%, *n* = 43), and 39.3% (*n* = 22) reported having two children. Nearly all parents were foreign-born (96.4%, *n* = 54), originating from four Central American countries—half were from El Salvador (50%; *n* = 27), 25.9% from Guatemala (*n* = 14), 22.2% from Honduras (*n* = 12), and 1.9% from Panama (*n* = 1). Foreign-born parents reported living in the US for an average of 13.7 years (SD = 7.2), and the vast majority (94.4%, *n* = 51) was categorized as having low acculturation levels (SASH score < 2.99) [34].

Furthermore, half of the study sample had completed less than high school education or obtained a high school diploma or General Education Degree (GED) (50%, *n* = 28). The vast majority was employed (96.4%; *n* = 54), and 78.6% (*n* = 44) reported annual household income earnings of less than US$40,000. The majority (89.3%, *n* = 50) reported having access to healthcare through either government-sponsored health insurance (e.g., MassHealth or Medicaid) (80.4.9%, *n* = 45) or private insurance (8.9%, *n* = 5). 

### 3.2. HPV and HPV Vaccine Awareness

**Figure 1:**
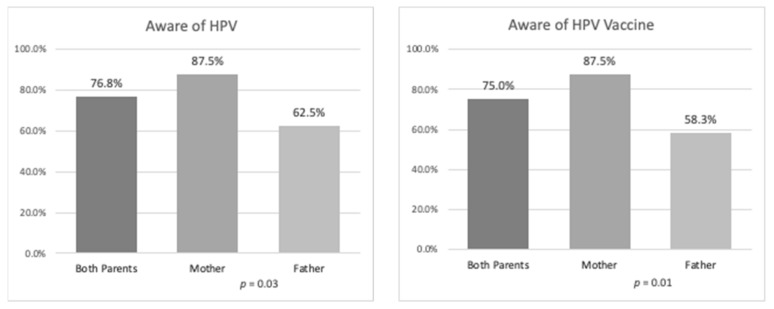
Central American parents’ awareness of HPV and the HPV vaccine (*n* = 56).

As shown in both Figure 1 and Table 2, 76.8% (n = 43) of parents were aware of HPV. Fathers reported lower awareness of HPV than mothers (62.5%, n = 15 versus 87.5%, n = 28; p = 0.03) (Table 2).

Although awareness of HPV was lower among parents with low acculturation levels (SASH < 2.99; 80.4%, n = 41 versus 66.7%, n = 2; p = 1.00) and those who reported household income less than US$40,000/year (75%, n = 33 versus 100%, n = 10; p = 0.08) than among parents with high acculturation levels (SASH ≥ 2.99) and parents who reported household income more than or equal to US$40,000/year, these differences were not statistically significant (see Table 3). In contrast, as shown in Table 3, awareness of HPV was significantly lower among parents with low educational attainment (less than high school/diploma or GED; 53.6%, n = 15 vs. 92.9%, n = 26; p = 0.01) than among parents with high educational attainment (high school/diploma or college). 

Awareness of the HPV vaccine was slightly lower, with 75% (n = 42) of parents reporting they had heard of the HPV vaccine (Table 2 and Figure 1). Fewer fathers had heard of the HPV vaccine than mothers (58.3%, n = 14 versus 87.5%, n = 28; p = 0.01) (Table 2). Although awareness of the HPV vaccine was lower among parents with low acculturation levels (76.5%, n = 39 versus 66.7%, n = 2; p = 1.00) than those with high acculturation levels (SASH score > 2.99), this difference was not statistically significant (Table 3). In contrast, as shown in Table 3, awareness of the HPV vaccine was significantly lower among parents with low educational attainment (i.e., less than high school or diploma; 53.6%, n = 15 vs. 92.9%, n = 26; p = 0.01) and parents who reported household income less than US$40,000/year (70.5%, n = 31 versus 100%, n = 10; p = 0.05) than those with high educational attainment (high school/diploma or college) and those who reported household income more than or equal to US$40,000/year (Table 3).

### 3.3. Sources of Information About the HPV Vaccine

Parents who were aware of the HPV vaccine (75%, n = 42) were asked where they heard about the HPV vaccine and could choose multiple sources of information (Table 2). As shown in Table 2, parents cited both healthcare system and non-healthcare system information sources, with healthcare system being the most common information sources reported by parents. Healthcare system information sources included child’s physician (83.3%, n =35), nurses (14.3%; n = 6), and public health campaigns (16.7%; n = 7). Fewer fathers than mothers reported hearing about the HPV vaccine from their child’s physician (57.1%, n = 8 vs. 96.4% n = 27; p = 0.01). In contrast, more fathers than mothers reported hearing about the HPV vaccine from public health campaigns (35.7%, n = 5 versus 7.1%, n = 2; p = 0.02).

Non-healthcare information sources included personal social network members such as family (23.8%; n = 10) and friends (4.8%, n = 2), and media sources such as the internet (23.8%, n = 10), media campaigns on the TV and the radio (19%, n = 8), and billboards (4.8%, n = 2). As shown in Table 2, more fathers reported hearing about the HPV vaccine from family members (50%, n = 7 versus 10.7%, n = 3; p = 0.005), and from media campaigns on the TV and the radio (42.9%, n = 6 versus 7.1%, n = 2; p = 0.06) than mothers.

Furthermore, parents who reported being aware of the HPV vaccine (75%, n = 42) were asked if their child had received the HPV vaccination, and of these, 85.7% (n = 36) reported their children had received at least one dose of the HPV vaccine. Fewer fathers than mothers reported their children had received the HPV vaccine (64.3%, n = 9 versus 96.4%, n = 27; p = 0.06) (Table 2). 

### 3.4. Acceptability of the HPV Vaccine and Willingness to Vaccinate

Moreover, parents who reported they had not heard of the HPV vaccine (n = 14); or that their children had not received the HPV vaccine (n = 1); or who were not sure if their child had received the HPV vaccine (n = 5), were asked whether they would be willing to have their children vaccinated if recommended by their child’s physician. Ninety percent (n = 18) of these parents reported they would be willing to vaccinate their age-eligible children against HPV if recommended by their child’s physician, whereas 10% (n = 2) reported they were not sure (Table 2). Although willingness to vaccinate with physician’s recommendation was lower among fathers than mothers (86.7%, n = 13 vs. 100%, n = 5; p = 1.00), this difference was not statistically significant.

### 3.5. Interest in Participating in Future HPV-Related Cancer Prevention Study

Finally, when asked about their interest in participating in future HPV-related cancer prevention study, the majority of parents reported being interested (87.5%; n = 49) (Table 2). Although mothers reported higher interest in participating in future HPV-related cancer prevention study than fathers (90.6%; n = 29 versus 83.3%, n = 20; p = 0.42), this difference was not statistically significant.

## 4. Discussion

To our knowledge this pilot cross-sectional study is the first study conducted in the US assessing Central American immigrant parents’ awareness, acceptability, and willingness to vaccinate their adolescent children against HPV. In addition, this study adds to a dearth of research assessing Latino fathers’ awareness of HPV and the HPV vaccine [21,25,26,28]. In the present study, fathers made up about 43% of the total sample, a higher representation than that of previously studies (from 8.3% to 31.9% of the sample) conducted with Latinos in the US [14,15,16,17,21,22,23,24,25,26,27].

Study findings found that 76.8% of parents had heard of HPV and 75% had heard of the HPV vaccine. These findings indicate that although awareness of the HPV vaccine in this group of Central American immigrant parents is lower than the target level set by the Healthy People 2020 (80%) [12], it is higher than that reported for the full US population (66%) [38]. Nevertheless, this study found that awareness of HPV (62.5%) and the HPV vaccine (58.3%) was significantly lower among fathers than mothers. Prior studies conducted in the US have also found awareness of HPV and the HPV vaccine among other racial/ethnic parents to be lower than the 80% target level set by the Healthy People 2020 [38,39,40,41]. Moreover, similar to our findings, these prior studies also documented that mothers are more likely than fathers to be aware of HPV and the HPV vaccine [38,39,40,41]. Additionally, our study findings are comparable to a limited number of studies conducted with other Latino groups that have found fathers are less aware of HPV and the HPV vaccine than mothers [21,26]. For example, a cross-sectional study conducted in Utah with Latino parents, 14.5% of which were fathers and majority immigrants from Mexico, found that fathers’ awareness of the HPV and the HPV vaccine was significantly lower than mothers [21]. Similarly, a cross-sectional study conducted in Puerto Rico with parents who had at least one son, 11.5% of which were fathers, found that 62.4% had heard about the HPV vaccine [26]. Likewise, a cross-sectional study with a national sample of Latino fathers, most of whom were foreign-born (the majority were from Mexico) found that among those who were HPV-aware, only 52.7% had heard of the HPV vaccine known as Gardasil [25]. Findings of our study taken together with that of previous studies suggest the need for efforts targeting fathers to increase fathers’ awareness of HPV and the HPV vaccine [21,26].

Furthermore, the present study found that awareness of the HPV vaccine was significantly lower among parents with lower educational attainment (less than high school or diploma) than those with higher educational attainment (high school/diploma or college). Study findings also showed that awareness of the HPV vaccine was statistically significant lower among parents who reported household income of less than US$40,000/year than those who reported household annual income of more than or equal to US$40,000/year. Our findings are consistent with previous studies conducted among Latino parents [42,43,44,45] as well as other racial and ethnic minority groups in the US [18,38,39,40,41,42,46,47,48,49,50,51]. These findings, combined with the fact that nearly all parents participating in our study were classified as having low acculturation levels (SASH <2.99), suggest that interventions designed for Central American immigrant parents should take into account parents’ educational and acculturation levels, and include culturally and linguistically tailored messages and materials presented at an accessible reading level to meet the specific needs of this ethnic minority group.

The majority of parents (83.3%) participating in this study reported having heard of the HPV vaccine from their child’s physician. This important finding adds to research evidence that indicates the central role physician’s play in increasing parents’ awareness of the HPV vaccine, and ultimately in influencing the uptake of the HPV vaccine for their adolescent children [52,53,54]. Nonetheless, in agreement with prior studies conducted among Latino parents [21,26,28,29] and other racial/ethnic minorities [18,38,39,40,41], findings of the present study also determined that fewer fathers reported hearing about the HPV vaccine from their child’s physician than mothers. In contrast, more fathers than mothers reported hearing about the HPV vaccine from public health campaigns, media campaigns on the TV and the radio, and family members. Although more research is needed, these findings have implications for the design of educational efforts targeting Central American immigrant fathers. For example, these findings suggest that culturally sensitive public health campaigns using the TV and the radio, as well as educational interventions that include other family members might be suitable channels to reach Central American fathers and increase their awareness of the HPV vaccine, as fathers may be less likely than mothers to be the parent to take their child to healthcare visits. 

A notable finding of this study is that among parents who were aware of the HPV vaccine (75%, *n* = 42), the majority (85.7%, *n* = 36) reported their children had received the HPV vaccine. Although more research is needed, this finding suggests that increasing awareness of the HPV vaccine would likely increase Central American parents’ acceptability of the vaccine for their age-eligible adolescent children. Despite that, findings also showed that fewer fathers reported their children had received the HPV vaccine (64.3%, *n* = 9 versus 96.4%, *n* = 27; *p* = 0.06) than mothers. This finding concurs with that of a limited number of studies conducted with other racial/ethnic parents [18,38,39,40,41] and Latino immigrant parents that determined that fathers are less likely to report their children uptake of the HPV vaccine [21,26]. For example, a cross-sectional study conducted in Puerto Rico with Puerto Rican and Dominican parents that found that fathers were 88% less likely to report that their sons had initiated the HPV vaccine regimen (Odds Ratio (OR) = 0.12; 95% CI = 0.02–0.90) compared with mothers [26]. Our finding adds to the scant literature on Latino fathers [21,25,26], and as previously noted, suggests the need for intensified educational efforts to increase awareness and acceptability of the HPV vaccine among Latino fathers, which likely would contribute to increased vaccine uptake among their age-eligible children.

Notably, the majority of parents (90%, *n* = 18) participating in this study whose children were unvaccinated or who were unsure whether their children had received the HPV vaccine reported willingness to vaccinate if recommended by their child’s physician. In spite of this, study findings also determined that fewer fathers (86.7% versus 100%, *p* = 1.00) reported willingness to vaccinate if recommended by their child’s physician than mothers. Although this difference was not statistically significant, this might be influenced by the small sample size, suggesting the need for future studies with a larger sample size to further examine this difference in parental willingness to vaccinate their children against HPV. Nonetheless, our study’s findings coupled with evidence from prior research documenting lower acceptability of the HPV vaccine among fathers than mothers [18,21,25,26] underscore the importance of renewed efforts to intensify physicians’ discussion and recommendation of the HPV vaccine, and the importance of increased focus on fathers, which combined can significantly influence Latino parents’ decisions to vaccinate their children.

Finally, the majority of parents (87.5%, *n* = 49) participating in this study reported high interest in participating in future HPV-related cancer prevention study. Although fathers reported lower interest in participating in future HPV-related cancer prevention study than mothers (83.3%, *n* = 20 versus 90.6%; *n* = 29; *p* = 0.42), this difference was not statistically significant. This finding is important and suggests the need for and the viability of future HPV-related cancer prevention studies given parents’ receptivity to information about the HPV vaccine, the lack of research among Central American immigrant parents, and in particularly, the dearth of research among Latino immigrant fathers living in the US.

We acknowledge that this pilot cross-sectional study has several limitations. First, findings are based on a small, convenience sample of Central American immigrant parents living in a few cities in MA and RI, which limits study generalizability beyond the sample population [55]. In addition, as is often the case with formative and non-random exploratory designs [56], the small sample size limits our ability for more sophisticated analysis including accounting for potential confounding factors. Participating parents may have had a heightened interest and awareness regarding the study topics. Snowball sampling also might have resulted in recruiting study participants who share similar awareness of HPV and the HPV vaccine. Thus, further research is needed to increase generalizability and to explore whether results apply to a broader group of Central American immigrant parents. Furthermore, this study assessed only limited information about Central American parents’ awareness of HPV and the HPV vaccine. Therefore, future studies should consider all these limitation and build on the findings of this exploratory, pilot study by having a larger sample size, employing other research designs and multiple data-collection methods including both qualitative and quantitative methods, and measuring and assessing the effects of additional variables using multivariate analyses. 

## 5. Conclusions

Despite limitations, this pilot study contributes to building an understanding of Central American immigrant parents’ awareness, acceptability, and willingness to vaccinate their children against HPV if recommended by a child’s physician. Findings are particularly important given the dearth of information specific to Central American immigrant parents, and in particular, the dearth of research on Latino fathers. This information is important and needed to develop culturally relevant educational and prevention strategies for Central American immigrant parents aimed at improving HPV vaccination rates among their age-eligible children, and, thereby decreasing their HPV-associated cancer risk and cancer health disparities. 

## Figures and Tables

**Table 1:** Characteristics of the sample (*N* = 56).

Variables	Total Sample			Fathers			Mothers		
	N	%	M (SD)	N	%	M (SD)	N	%	M (SD ^*^)
**Socio-demographic variables**									
**Age**	56		43.2 (6.4)	24		39.6 (8.2)	32		45.8 (7.3)
**Educational attainment**									
Less than high school	28	50.0		14	58.3		14	43.8	
High school/diploma (GED) ^1^	18	32.1		7	29.2		11	34.3	
More than high school	10	17.9		3	12.5		7	21.9	
**Marital Status**									
Married/Cohabitating	43	76.8		19	79.2		24	75.0	
Divorced/separated	10	17.9		4	16.7		6	18.7	
Single	3	5.2		1	4.1		2	6.3	
**Number of children 11–19 years of age**									
1	32	57.1		13	54.2		19	59.4	
2	22	39.3		10	41.7		12	31.5	
3	2	3.6		1	8.3		1	3.1	
**Annual household income ^2^**									
Less than US$40,000/year	44	78.6		15	62.5		29	90.6	
More than or equal to US$40,000/year	10	17.9		7	29.2		3	9.4	
Missing	2	3.5		2	8.3		0	0	
**Health insurance status**									
Government health insurance	45	80.4		15	62.5		30	93.7	
Private health insurance	5	8.9		5	20.8		0	0	
Uninsured	6	10.7		4	16.7		2	6.3	
**Foreign-born**									
Yes	54	96.4		23	95.8		31	96.8	
No	2	3.6		1	4.2		1	3.2	
**Country of Origin of foreign-born**									
El Salvador	27	50.0		13	56.6		14	45.2	
Guatemala	14	25.9		5	21.7		9	29.0	
Honduras	12	22.2		5	21.7		7	22.6	
Panama	1	1.9		0	0		1	3.2	
**Acculturation variables**									
**Years of residence in the United States**	54		13.7 (7.2)	23		15.8 (8.9)	31		12.9 (9.4)
**SASH score ^3, 4^**									
<2.99	51	94.4		21	91.3		30	96.8	
≥2.99	3	5.6		2	8.7		1	3.2	

* Standard Deviation; ^1^ HS diploma = General Education Degree (GED); ^2^ Family of 4 household income <US$40,000/year is consider low-income in Massachusetts; ^3^ SASH: Short Acculturation Scale for Hispanics; ^4^ The scale developers recommend an average of ≥ 2.99 as the cut point—scores equal to or above this point represent higher levels of acculturation and scores below this point represent lower levels of acculturation.

**Table 2:** Central American parents’ awareness of HPV and the HPV vaccine, acceptability and willingness to vaccinate against HPV, and interest in participating in HPV-related cancer prevention study (*n* = 56).

Survey Measures	Total Sample		Fathers		Mothers		*p*-Value ^1^
	N	%	N	%	N	%	
**Awareness of HPV**							
Yes	43	76.8	15	62.5	28	87.5	*p* = 0.03
No	13	23.1	9	32.5	4	12.5	*p* = 0.03
**Awareness of the HPV vaccine**							
Yes	42	75.0	14	58.3	28	87.5	*p* = 0.01
No	14	25.0	10	41.7	4	12.5	*p* = 0.03
**Sources of information about the HPV vaccine ^2 ,3^**							
**Health care information sources**							
Child’s physician	35	83.3	8	57.1	27	96.4	*p* = 0.01
Nurses	6	14.3	2	14.3	4	14.3	*p* = 0.96
Public health campaigns	7	16.7	5	35.7	2	7.1	*p* = 0.02
**Non-health care information sources**							
Personal sources							
Family	10	23.8	7	50.0	3	10.7	*p* = 0.05
Friends	2	4.8	0	0	2	7.1	*p* = 0.54
**Media sources**							
Internet	10	23.8	4	28.6	6	21.4	*p* = 0.61
Television or radio	8	19.0	6	42.9	2	7.1	*p* = 0.06
Billboards	2	4.8	1	7.1	1	3.6	*p* = 1.00
**Acceptability of the HPV vaccine ^4^**							
Yes	36	85.7	9	64.3	27	96.4	*p* = 0.06
No	1	2.4	0	0	1	3.6	*p* = 1.00
Don’t know	5	12.2	5	35.7	0	0	*p* = 0.01
**Willingness to vaccinate if recommended by child’s physician ^5^**							
Yes	18	90.0	13	86.7	5	100	*p* = 1.00
No	2	10.0	2	13.3	0	0	*p* = 1.00
**Interest in participating in HPV-related cancer prevention study**							
Yes	49	87.5	20	83.3	29	90.6	*p* = 0.42
No	7	12.5	4	16.7	3	9.4	*p* = 0.42

^1^*p*-Values generated from Chi-square tests and Fisher’s Exact for cells <5. ^2^ Question asked only to parents who reported had heard of HPV vaccine (*n* = 35). ^3^ Respondents could choose multiple sources of information. ^4^ Question asked only to parents who had heard of the HPV vaccine (*n* = 35). ^5^ Question asked to parents who: (a) had not heard of the HPV vaccine, (b) reported child had not received; or (c) were unsure if child received HPV vaccine.

**Table 3:** Central American parents’ awareness of HPV and the HPV vaccine, and interest in participating in future HPV-related cancer prevention study by acculturation score, educational attainment, and household income (*n* = 56).

Survey Measures	SASH Score ^1^			Educational Attainment			Household Income ^2^		
	N (%)			N (%)			N (%)		
	<2.99	≥2.99	*p*-Value	<HS	≥HS	*p*-Value	<US$40k	≥US$40k	*p*-Value ^3^
**Awareness of HPV**									
Yes	41 (80.4)	2 (66.7)	*p* = 1.00	15 (53.6)	26 (92.8)	*p* = 0.001	33 (75.0)	10 (100.0)	*p* = 0.08
No	10 (19.6)	1 (33.3)	*p* = 1.00	13 (46.4)	2 (7.2)	*p* = 0.001	11 (25.0)	0 (0)	*p* = 0.08
**Awareness of the HPV vaccine**									
Yes	39 (76.5)	2 (66.7)	*p* = 1.00	15 (53.6)	26 (92.8)	*p* = 0.001	31 (70.5)	10 (100.0)	*p* = 0.05
No	12 (23.5)	1 (33.3)	*p* = 1.00	13 (46.4)	2 (7.2)	*p* = 0.001	13 (29.5)	0	*p* = 0.28
**Uptake of the vaccine ^4^**									
Yes	33 (86.8)	3 (100.0)	*p* = 1.00	11 (73.3)	25 (96.2)	*p* = 0.03	26 (86.7)	10 (100.0)	*p* = 0.56
No	5 (13.2)	0 (0)	*p* = 1.00	4 (26.7)	1 (3.8)	*p* = 0.03	4 (13.3)	0	*p* = 0.56
**Willingness to vaccinate if recommended by child’s physician ^5^**									
Yes	15 (88.2)	0		13 (90.0)	3 (100.0)	*p* = 0.58	15 (88.2)	0	
No	2 (11.8)	0		4 (10.0)	0 (0)	*p* = 0.58	2 (11.8)	0	

^1^ SASH acculturation score calculated only for foreign-born parents (*n* = 54). ^2^ Two parents had missing values for household income (*n* = 54). ^3^ *p*-Values generated from Chi-square tests and Fisher’s Exact for cells <5. ^4^ Question asked only to parents who reported had heard of the HPV vaccine. ^5^ Question asked to parents who: (a) had not heard of the HPV vaccine, (b) reported child had not received; or (c) were unsure if child had received HPV vaccine.

## Abbreviations

The following abbreviations are used in this manuscript.

US	United States
HPV	Human Papillomavirus
MA	Massachusetts
RI	Rhode Island
GED	General Education Degree
SASH	Short Acculturation Scale for Hispanics
